# The Last of England: Banal Nationalism and Communities of Loss in British Pub Closure Media Narratives

**DOI:** 10.1111/1468-4446.13220

**Published:** 2025-05-07

**Authors:** Robert Deakin, Thomas Thurnell‐Read

**Affiliations:** ^1^ Loughborough University Loughborough UK

**Keywords:** closure, community, loss, national identity, nationalism, pubs

## Abstract

While pubs have long been celebrated as a quintessential part of British culture, the ongoing and increasingly rapid closure of British pubs has raised concerns about the impacts of their loss on the wider cultural life and identity of the nation. The article explores how pub closures are narrated in British print news media through the analysis of a sample of news stories spanning 2000–2023. Time series analysis shows that pub closures have been a steady concern in UK print media, albeit with several notable peaks in coverage aligned to key events impacting the sector. Findings suggest that the causes of pub closure are presented as an economic issue, while the consequences of pub closures are typical framed in social and cultural terms. Using Billig's concept of ‘banal nationalism’, the article analyses a sub‐set of this data to examine how the narratives used to explain pub closures make regular and emotive reference to the nation and associated concepts. Pub closures are therefore presented as a threat to the nation and a loss of national identity. These emotive narratives of loss, we argue, work to homogenise both the idealised pub and the wider national community in a manner which occludes the complexity of both.

*When you have lost your inns, drown your empty selves, for you will have lost the last of England.*

Hilaire Belloc, 1912

*A village without a pub is like a scone without clotted cream, a bank holiday without rain, Bake Off without the innuendo and a crucial football match without penalties. It just isn't British.*

Hugh Graham, The Times, 2021

## 1 Introduction

The public house (or ‘pub’) is an enduring institution in Britain. More than simply a space for consuming alcohol, pubs are hailed as places of significant social, economic and cultural importance (Jennings 2007). They are also frequently regarded as a ‘bastion of English culture’ (Everitt and Bowler 1996, 102), meaning pubs and pub going are ‘deeply ingrained in the English psyche’ (Haydon 1994). However, a long‐term trend has been the decline in pub numbers, with the last 2 decades seeing a net loss of over a quarter of Britain's pubs (BBPA, n.d.). While the ‘death’ of the English pub has long been forewarned (Hutt 1973), this seemingly inexorable decline has seen pubs and pub closure become an object of concern and anxiety within British media and cultural politics. This article analyses how pub closures operate as a site of ‘imagined community’ (Anderson 1991) in British print news media, with their closure tied up with expressions of national loss and decline that we formulate in terms of ‘banal nationalism’ (Billig 1995).

Recent sociological writing has highlighted the persistence of the cultural mythology of pubs (Markham 2024) and concerned the contemporary representation of the pub as a site of ‘idyllic English sociability’ and ‘working class authenticity’ under threat (Singh et al. 2024). Amid a wave of English nationalism, most obviously evidenced by the ‘Brexit’ vote (Calhoun 2017; Dodd et al. 2017), declinist representations of the pub are central to dominant and racially exclusionary imaginations of the nation. Indeed, there is statistical evidence that the loss of pubs is a material factor in support for far‐right political parties (Bolet 2021). Contemporary representations of the pub as a site of white, working‐class nationhood sit alongside and draw upon longer imagined associations between the pub and the nation in which the rural village origins of the ‘traditional English pub’ are emphasised (Kingsnorth 2008; Markham 2014).

While the British pub, and British pub culture more generally, are frequently invoked in narratives of nationhood and national belonging, there has yet to be a comprehensive survey of how such claims are made and sustained and, importantly, where lines of inclusion and exclusion are drawn. This article pursues this through an empirical analysis of UK print media articles on pub closure between 2000 and 2023. This period spans various developments and events associated with accelerating pub closures, including the 2007 smoking ban, the 2008–9 global economic recession, as well as longer terms shifts to home drinking and reduced alcohol consumption, especially amongst young people (Snowdon 2014). As will be shown, media pub closure narratives persistently represent the issue of pub closure as a threat to British or English national belonging, culture and heritage. Rather than being the province of a specific aggrieved white, working class—as per Singh et al. (2024)—we find that invocations of the pub as a site of national community attempt to rhetorically transcend class and other social divides. We argue that the link between the pub and nation in these media texts is best grasped through Billig's (1995) concept of ‘banal nationalism’. Pub closure narratives serve to ‘banalise’ the issue of pub closure by (i) occluding the heterogeneity that exists within the British pub landscape and (ii) positioning as universal what is, in practice, a particular space that often caters to some social groups rather than others. It is therefore not only the nation which is reproduced through these media narratives, but a normative conception of the pub most compatible with it.

## 2 Locating the Pub in the National Cultural Imagination

The pub is central to representations of communal life in Britain (Furnham et al. 1997). Indeed, pubs have long been held in the public imagination as important public spaces, representative of concerns about collective rituals and solidarities (Kneale 2001). During the 20th century they became part of popular culture through representation in mass media and through the expansion and standardisation of an increasingly commercialised pub sector (Everitt and Bowler 1996). These popular representations stretch across urban, suburban and rural geographies. For example, the urban and suburban neighbourhood pub of industrial British cities is central to the storytelling within popular long‐running TV soap operas such as *EastEnders* and *Coronation Street* ^1^ and ‘The Woolpack’ pub is a key setting within *Emmerdale*, set in rural Yorkshire. Across these soap operas, the pub is a stage for dramatic events to unfold but is also presented as the archetypical ‘third place’ (Oldenburg 1989), a space other than home or work where a community can be formed and maintained through co‐presence and quotidian, binding, social interactions (Thurnell‐Read 2021a).

In line with these dominant representations, sociological work has explored pubs as important sites of social connection, belonging and community (Dunbar 2016; Thurnell‐Read 2021b, 2024). These attributes are sometimes conceptualised and quantified in terms of ‘social capital’ whereby the presence of pubs is correlated with measures of socio‐economic development and social cohesion (Cabras and Mount 2017; Sforzi and Bianchi 2020; Author, D). This work exists alongside, and somewhat in tension with, other literature which has stressed the ways pubs have always been sites of exclusion and distinction around class, gender, race and sexuality. The pubs of the late 19th and early 20th century were commonly organised into separate spaces, such as the ‘public bar’ and the ‘lounge’, the former excluding women and the latter enforcing dress codes and more respectable conduct from its patrons (Hey 1986; Jennings 2007). While a notable trend in the second half of the 20^th^ century was the physical removal of such partitions, social hierarchies and exclusions are sustained, for example through the snobbery directed at certain ‘types’ of pubs such as the ‘flat‐roofed’ pubs of municipal housing estates (Boak and Bailey 2017) and in terms of the pubs that women and sexual minorities feel most welcome in (Campkin 2023). Pubs have also been sites of hierarchy and exclusion around race. Research in this area has evidenced the ‘colour bars’ denying Black and Asian migrant communities entry to certain pubs in the post‐war period, the struggles for inclusion by those excluded, and the establishment of alternative drinking spaces catering to minority communities (Jesudason 2023; Hirsch and Brown 2023; Singh et al. 2024).

Such critical perspectives on pubs, and the types of communities they serve and sustain, are all the more necessary in a contemporary context where the pub—and the issue of pub closures—is cast in national and nationalistic terms. Pubs are sites of everyday consumption, but they are also richly symbolic spaces where the beer people drink invokes nostalgia for pastoral rural pasts and an age of industrial and martial Imperial dominance (Thurnell‐Read 2015). Matless (2023), for instance, notes the zeal with which many public figures, notably the right‐wing politician Nigel Farage, have staged their political persona in the space of the pub and how rural pubs were a recurring image in materials produced by UKIP in the build up to and during Brexit. (Although, as Rankine et al. (2023) have recently highlighted with respect to former leader of the Scottish National Party, Nicola Sturgeon, it is not only politicians on the political right that have sought to use the pub in the service of national populist political messaging. Indeed, to be photographed in a pub, either drinking or pulling a pint, is almost obligatory for all British politicians during general election campaigns). Meanwhile for Singh et al. (2024), it is through the figure of a ‘left behind’ white, working class that the issue of pub closures has most significant resonance within contemporary cultural politics. The authors point to comments made by prominent politicians in 2015 blaming pub closures in ‘traditional working class areas’ on Muslim immigration and the subsequent enrolment of the pub within pro‐Brexit political campaigning. Here the pub is cast as an icon of English culture and heritage and then readily ‘lamented as under threat and in need of saving, as is the white working‐class pub goer who is seen as its exemplary patron’ (Singh et al. 2024, 2).

Such perspectives point to important connections between the representation of pub closure in the UK and contemporary articulations of race, nation and nationalism. But more remains to be understood about the myriad linkages between media representations of pubs and invocations of community and nation. In particular, there are perhaps more subtle and banal ways in which these links are made which, contra Singh et al. (2024), do not rest upon explicit representations of the closed pub as the domain of a particular social group such as the ‘white working class’. If Singh et al. (2024, 10) offer a ‘conjunctural analysis’ of the place of the pub within the ‘particularities of a post‐neoliberal new right populism’, we present a broader survey of the relationship between the pub and the ‘banal nationalism’ of British pub closure media narratives since the turn of the millennium.

In his now classic text *Banal Nationalism*, Billig (1995) draws a contrast between what he terms ‘hot’ and ‘banal’ forms of nationalism. The former denotes forms of nationalism which are overtly labelled as such and often considered characteristic of the peripheries or the extremes, such as far right political parties or separatist groups. Yet Billig argues that nationalism is a dominant ideology of Western liberal nations too, but is often so ‘banal’ and everyday as to remain unnoticed. Billig's intercession in the debates about nation and nationalism have been profoundly influential (Antonsich and Skey 2017) and firmly placed the banal reproduction of the nation on the agenda of scholars from different disciplines and regions (Koch and Paasi 2016). The significance of everyday rituals and practices to sustaining a sense of national identity and belonging is something Skey (2013: 94) has highlighted by suggesting that ‘national forms of life, realized through everyday practices, talk, material objects and spatio‐temporal arrangements, underpin a meaningful and stable sense of ‘ordered reality’ which ‘may become valued because it informs an ongoing and consistent sense of self, community and place’. As well as being of continuing relevance to nationalism studies, the concept of banal nationalism has also been employed to grasp how nationalist imaginaries define the stakes of other issues—such as sexual violence in the military and college campuses in the USA (Christian et al. 2016).

Billig (1995) himself draws attention to the pub as a topic of banal nationalism in a survey of daily national newspapers from 1993. He considers a special supplement ‐ *The Great British Pub—*included in *The Mirror* newspaper as part of its celebration of ‘British Pub Week’ (Billig 1995, 114). This celebration of a ‘bastion of British social life’ is an excellent example of banal nationalism for Billig: ‘From the title onwards, the supplement promoted heritage themes, waving flags and the national first‐person plural’ (ibid, 114). Banal nationalism operates here, by occluding the pub's exclusivity. Thus, Billig observes that the Great British Pub supplement addresses a specifically male readership yet does so through the presentation of the pub, in the most general terms, as “our” institution, “our” old tradition, a home from home for all of “us”. Here the repeated use of the first‐person plural makes ‘the part [represent] the whole’ (Billig 1995, 119). Billig further highlights how banal nationalism operates not through the creation of a nationalistic spectacle, but through something ordinary and unremarkable. Thus, while the spurious invention of ‘British Pub Week’ was itself ‘fated to be unmemorable’, its significance for Billig lay in it being illustrative of a repeated ‘flagging’ of the nation through such news items (particularly the sports pages) that banal nationalism works to become a background, but not benign, feature of daily life.

In what follows, we pick up Billig's important but until now overlooked concern with the ways in which banal nationalism works to reinforce a normative conception of the pub as symbolic of national culture and character. In the next section we present our research methods, outlining the sampling and analysis of British print media coverage of pub closures. We then present findings in two sections: the first addressing quantitative patterns and trends and the second exploring qualitative narratives. These findings are subsequently unpacked in the discussion section in which we reflect on how pub closure media narratives work to occlude the ‘heterogeneity’ of pubs in Britain (Maye et al. 2005), the varied ways pub closure is experienced, and index the strong sympathy that is afforded to the issue of pub closure in UK print media as a symbol of national culture under threat.

## 3 Methods and Context

The empirical material that we draw on in this article derives from a content analysis of 598 news items regarding pub closure published in the UK between 2000 and 2023, followed by in‐depth qualitative analysis of a smaller sample of articles. This time frame was selected to span a range of events and developments said to have a specific impact on the pub trade (the Licensing Act 2003; the smoking ban 1st July 2007; the global economic recession of 2008–9; the Covid pandemic and subsequent cost of living crisis). An initial sample of 2664 news items was generated using the Nexis database by searching for keywords ‘pub’ and ‘closure’. To eliminate false positives associated solely with the temporary measures forcing pubs to close in during the Covid pandemic, all articles containing the term “COBRA” (the acronym for Cabinet Office Briefing Rooms—the government entity which coordinates responses to national crises such as the covid‐19 pandemic) were excluded, along with those which included the terms ‘lockdown’, ‘covid’, ‘coronavirus’, ‘social distancing’ in the headline or lead paragraph of the article. Further restrictions were made to bring the sample down to a more manageable size for coding, retaining only: those articles which mentioned the word ‘closure’ in the headline or lead paragraph of the article, contained at least three mentions of the word ‘pub’, and at least two mentions of the term ‘closure’, and included the term pub within at least three words of the word closure. This resulted in a sample of 817 news items from a mixture of regional (67%) and national newspapers (33%). This sample included newspapers from all of the UK's constituent nations except for Northern Ireland.

A coding manual was created in order to capture: item size, date, name of newspaper, genre of article, section of newspaper, regional focus, type of pub under threat, geography (rural, suburban, urban), the 3 most prominent themes regarding causes of pub closure, the 3 most prominent themes regarding consequences of pub closure, the social group most affected (if any), and details of up to 5 actors appearing in the article—type of actor (e.g. publican, customer, politician), actor gender, and whether the actor was directly quoted. An initial pilot was attempted on a sample of 20 items. While coding the first 150 items it was found necessary to add and adjust variables where important themes and actors were encountered which had not been included within the coding manual. 598 news items were ultimately coded after filtering out duplicates (e.g., where the same story had been syndicated across several local newspapers) and other false positives (such as items relating to a pub being one of several businesses temporarily closed due to flooding). The data generated through coding was then analysed using SPSS to determine key trends and narratives (discussed below). Nine main narratives regarding pub closure were ascertained guided by the SPSS data and the researcher's experience of having read all the articles in the sample. A threshold of 10 example articles was set to sufficiently evidence the existence of an identifiable narrative. 122 articles then formed the sample for a more in‐depth qualitative analysis.

## 4 Content Analysis Findings

### 4.1 Time Series

**FIGURE 1:**
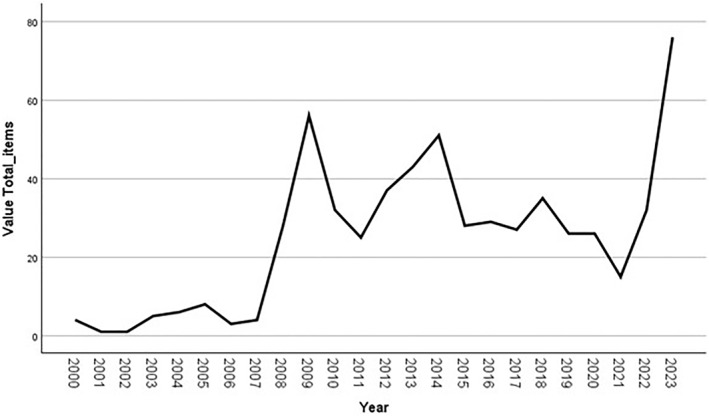
Time series distribution of pub closure press coverage.

Figure 1 shows that while news items about pub closures are a long‐term fixture in UK print media, there are noticeable spikes in coverage from 2008 onwards. This includes 2009 (56 articles), when the UK economy was in recession after the 2008 financial crisis, and 2014 (51 articles), when significant reforms to the ‘beer tie’ governing pub ownership structures were being debated in the UK parliament. Meanwhile, only 15 news items were coded from 2021, perhaps due to a tendency for the media to focus on temporary closures due to the covid‐19 pandemic, and only 32 news items were coded from the years 2000–2007, a pattern possibly exaggerated by limitations within the Nexis archive for smaller regional newspapers during the earlier part of the sample period. Most recently, 76 articles were from 2023 alone, the last year of our sample coinciding with what was often framed as a ‘cost of living crisis’, representing 13% of the entire sample. Media interest in the issue of pubs closures appears to be correlated with challenging periods for the commercial prosperity of pubs. But the amount of media interest around pub closures did not match up with net pub closures per year. For example, in 2017 there was a net loss of nearly 1950 pubs—the highest net loss over the 23 year period—but far fewer articles on pub closure than in 2023, when there was a net loss of 500 pubs (BBPA, n.d..). It would appear therefore that the frequency of stories about pub closure is also related to other factors, perhaps coming to stand symbolically for a broader sense of crisis precipitated by events such as the 2008 global recession and 2023 energy crisis—a point we will return to later.

### 4.2 Causes and Consequences of Closure

**FIGURE 2:**
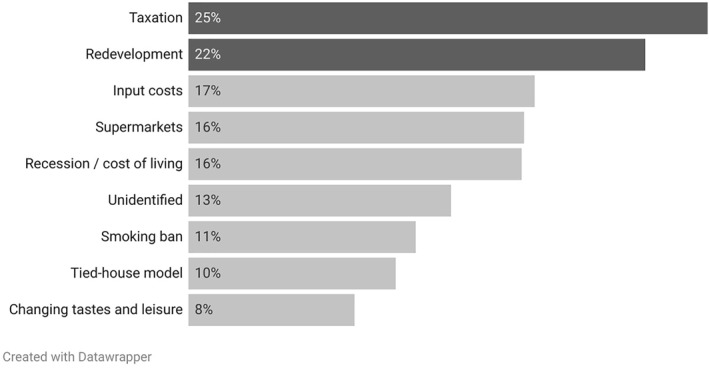
Top causes of pub closure by percentage of items.

**FIGURE 3:**
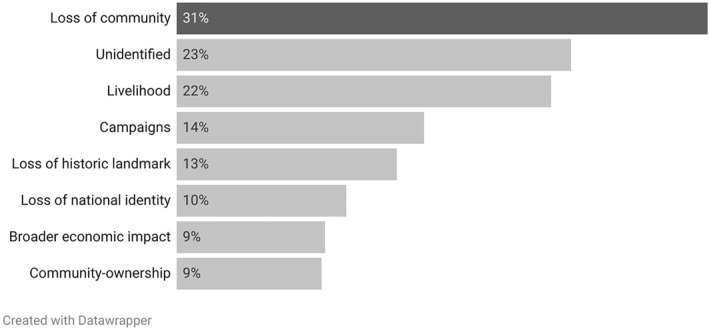
Top consequences of pub closure by percentage of items.

Having established that press coverage of pub closure underwent several spikes since the turn of the century, the next step in analysis was to examine the attribution of cause and consequences of pub closures. For each item the coding manual allowed for data to be collected on the top 3 causes and top 3 consequences of pub closure. These are shown in Figures 2 and 3 respectively.

Figure 2 shows the top 9 cited reasons for pub closure in the sample. There was a tendency for economic factors to be asserted as the causes of pub closure. Thus, the top three cited reasons for closure were excessive taxation (25%), redevelopment for residential use due to economic incentives (22%) and input costs (17%). Meanwhile, social and cultural explanations of pub closure, such as changing consumer tastes and leisure patterns (8%) and migration/demographic change (1.5%) were significantly less common. Such an emphasis on economic factors was heavily shaped by the centrality of trade bodies such as the British Beer and Pubs Association (BBPA) and pub campaign groups such as the Campaign for Real Ale (CAMRA) within reporting on pub closures. Both groups have run campaigns for government to increase financial support for pubs in the form of tax cuts and, in the case of CAMRA, to tighten planning laws to make it more difficult to convert pubs into residential properties. Here it is also worth noting how the BBPA drives some of the media interest around pub closures with its compilation and regular release of detailed statistics, often presented in an eye‐catching form such as the number of pubs which have been ‘lost’ per week over the last year.

Regarding the consequences of pub closure, Figure 3 shows how the relationship is reversed with social and cultural factors generally more prominent than economic factors. Thus, the loss of community and/or a place to socialise was by far the most discussed consequence of closure (31% of items), with campaigns against closure (14%), loss of historic landmarks (13%) and loss of national identity (10%) also prominent. Notably in relation to the themes of nationalism under consideration here, overt references to loss of national identity were more common (10%) than references to impact on local or regional identity or way of life (6%). On the economic side, loss of livelihood was the second most prominent theme overall (22%), with broader economic impacts in sixth place (9%). Thus, while press coverage might be seen as appreciative of the varied economic, cultural and social facets of the issue, the causes of closures lent towards the economic while the consequences of closure tended to speak to social and cultural matters.

### 4.3 Rural/Urban Comparisons

Given the noticeable skew towards rural pubs both in academic scholarship (Maye et al. 2005; Leyshon 2008; Cabras and Reggiani 2010; Markham 2024) and celebratory treatments in popular culture (Kingsnorth 2008), we expected to find a similar skew in our sample. We did not find a significant difference in the number of articles across geographies, with most (68%) not framing the issue of pub closures in terms of rural‐urban geography and, of those that did, 51% focusing on rural closures, 46% on urban closures and 6% on suburban closures. However, items focused on rural closures were significantly longer—31% on average—than those focused on urban closures. Meanwhile articles on rural closures were more likely to discuss the consequences of pub closure: only 8% of rural closure items contained no consequence theme compared to 28% of urban closure items. News articles on rural closures were therefore more likely to be the subject of long‐form, in‐depth articles which went into detail about the wider social issues connected to pub closures. In contrast, items focusing on the closure of pubs in urban areas tended to be shorter and more narrowly focused on the pragmatics of a given pub closing, such as economic struggles or the progress of planning applications for change of use to residential or retail space.

### 4.4 Actors, Pub‐Types and Social Groups Affected

It was possible to identify a clear pattern in who articles spoke of or spoke about. In keeping with sociological work which has analysed the gendered character of pub space and interaction (Nicholls 2017) (or, indeed, as Billig [1995: 119] noted pubs as ‘historically… masculine territory’), more than twice as many male actors appeared in the articles as women (482 vs. 203). Meanwhile, 82% of articles featured at least one male actor compared to only 34% for females. Looking at the first actor only, the discrepancy in gender representation was higher for national actors such as brewery/pub companies, pub campaigners and politicians, compared with local actors such as residents, customers and publicans.

The overwhelming majority (90% of articles) did not specify a social group that was most affected by pub closures. Among those that did, rural residents were most frequent (4%) followed by older people (2%). Relevant to the discussion about nationalism here, the issue of pub closures was very rarely explicitly framed around a working class or white British constituency (0.7% and 0.2% respectively). In contrast to research showing pubs to often be territorial spaces identified with a recognised clique of ‘regulars’ (Goode and Anderson 2015; Watson and Watson 2012), the impact of pub closures is depicted as being a national collective concern.

News items on pub closure frequently specified a particular ‘type’ of pub that was under threat. More than one‐third of articles (34%) used some form of prefix to indicate that the pub belonged to a particular locality or community—‘community pub’, local pub’, ‘village pub’ or ‘traditional pub’. Rather than merely being commercial enterprises, such pubs are seen as being within and in service of a locally specific, but vaguely defined, community. Meanwhile only 4% of items referred to chain pubs, 1% to gastro‐pubs and 0.3% to estate pubs, indicating a lack of understanding of or interest in the details of ownership structures and corporate operational strategies (Meers 2023) and reflecting prevailing hierarchies of which types of pubs are seen as worthy of cultural celebration (Boak and Bailey 2017). Importantly, for the narrative analysis that follows, coverage of pub closures appears to be most pronounced when the issue can be represented as impacting ordinary ‘Brits’ residing in ‘communities’ across the length and breadth of the nation and most readily encapsulated by the image of the traditional village pub. As we will come onto discuss, while this is presented as an inclusive image, it elides the ways that some types of people are more readily included as full members in this image of national community than others (Singh et al. 2024).

## 5 Narratives of Closure and Communities of Loss

**FIGURE 4:**
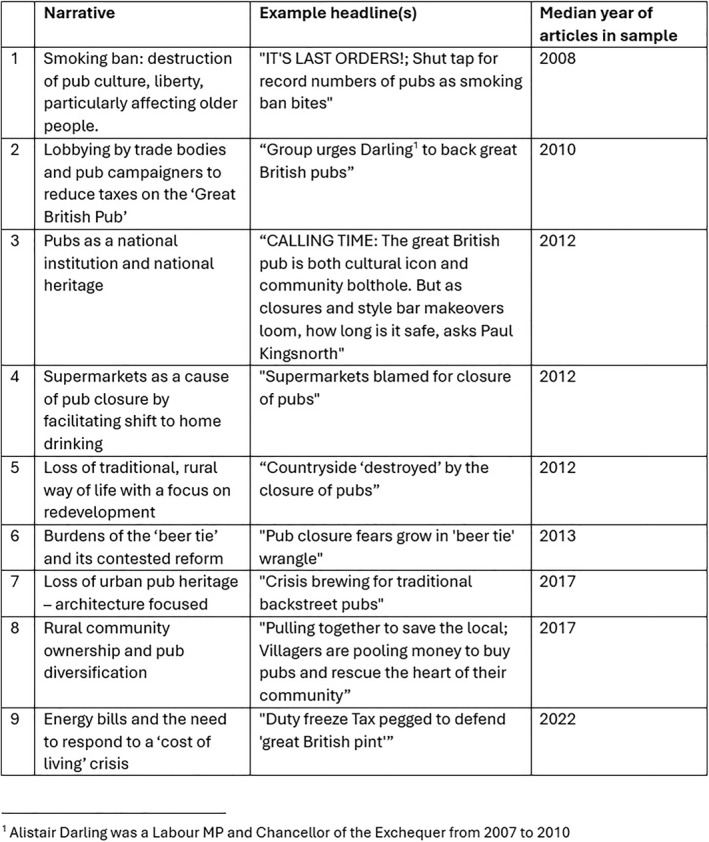
Qualitative narratives of pub closure.

The analysis of quantitative data derived from content analysis illuminates key patterns in the reporting of pub closures. Pub closures are presented as a topic of ongoing national concern, albeit with specific spikes in interest and several identifiable differences in how urban and rural pub closures are presented. Tellingly, across the coverage it is male voices, most prominently those representing corporate interests or campaign groups that are most frequently involved. Moving now beyond these general patterns, qualitative analysis of a focused sub‐sample of print media content was able to identify evocative narratives which recurred as a means of framing the debate about pub closures. Figure 4, below, lists 9 recurring narratives that were identified. Each narrative was substantiated with a minimum of 10 example articles meaning these 9 narratives were reoccurring and identifiable framings of the wider issue. They are listed in chronological order, based on the median year of these articles.

Across the 9 narratives the pub is presented as a traditional institution and a way of life under threat. The tone is broadly conservative, but different political philosophies can be inferred from how the threat to pubs is articulated and how it is asserted that these threats should be addressed. There is a tension between, on the one hand, a more libertarian demand for the state to leave pubs alone: for example, with a concern for the over‐regulation of pubs via the ‘smoking ban’ in narrative 1 and the concern with the over‐taxation of pubs in narrative 2. And, on the other hand, a more interventionist demand for the government to step in to save pubs which are at the mercy of private markets; whether in the form of competition from supermarkets (narrative 4), residential property conversion and development (narrative 5), extractive financial practices of pub companies (narrative 6), or a spike in energy costs (narrative 9). Across all the narratives one also finds a strong communitarian streak. This laments the loss of pubs as sites of community and, in the case of narrative 8, conveys a sense of optimism that community action, in the form of community‐ownership schemes, might save local pubs from closure.

While our content analysis found that loss of national identity was the 5th most prominently cited consequence of pub closure, a closer reading of the 122 article sample revealed the prevalence of references to national culture and identity. This is most clearly the case in the articles evidencing narrative 3, which explicitly position pubs as a site of national heritage. But even when these themes were not the most prominent in the article, and therefore did not necessarily register in the content analysis, particular words and phrases appeared to subtly shift the framing of other issues towards nation and national culture. For example, articles evidencing 7 out of the 9 narratives included the phrases ‘the Great British pub’ and/or ‘the Great British pint’. This is a form of ‘banal nationalist’ wordplay where the pub and the pint belong to, and stand as symbolic of, the country of Great Britain but are also positioned as part of what is assumed to make Britain a ‘great’ country in terms of its culture and values. While perhaps intended to be tongue‐in‐cheek, it still serves the purpose of reinforcing, in a banal and repetitious fashion, the link between pubs and the nation, with a corollary of this being the idea that the closure of Britain's pubs is at the same time a threat to the British nation itself.

This threat to the nation was conveyed in other ways. For example, in an article from the *Evening Chronicle* from 2012 focusing on the impact of supermarkets, a landlord of a recently closed pub in the northeast of England is quoted as saying that ‐ in forcing pubs to close ‐ supermarkets are ‘destroying the whole fabric of British community’. The article describes how the former landlord has placed posters in the pub windows criticising the government—‘Thanks [then British Prime Minister] Cameron, another pub closed’—and declaring that this amounts to ‘Losing our community, our identity, our culture, our heritage’. Similarly, an article in *The Mirror* from 2023 concerned with the loss of urban heritage, describes pubs as part of “*our* cultural heritage” (emphasis added), while an article in the *Financial Times* from 2012 on this theme defines pubs as a ‘British institution’. Following Billig's (1995) analysis of the role of deictic words in banal nationalism, we can see here how the repeated use of the possessive determiner ‘our’ in this context—‘our pubs’, ‘our heritage’—works to naturalise the connection between the person speaking, the people addressed and the object ‘pubs’ as all part of the same British community. As the first of these examples illustrates particularly acutely, the language deployed allows a ready leap from the particular, the closure of a specific pub in the northeast of England, to the general, the loss of a constituent part of the nation's identity that, by implication, the nation in its entirety can be impacted by and should care about. This is all while simultaneously obscuring the ways that, in practice, full membership of this community—and the privileges that bestows ‐ is delimited by social differences such as race and gender (Singh et al. 2024).

Across the nine narratives there is a braiding of themes of pubs, community, loss and nation. Articles using the 2007 ban on smoking in enclosed public spaces allowed the press to construct a narrative framing a way of life and British past time as being under threat. An article in the *Coventry Evening Telegraph* from 2008 quoted a publican of a closed pub:

Like it or not, Britain has a pub culture. People have enjoyed having a pint and a fag in a pub for generations. Now the government has changed all that. They're taking away the fabric of the British way of life.

Significant here is the way that the pub is used to mediate across both chronologies—from the closure in the present moment to the continuity represented by a history going back ‘for generations’—and different geographical scales—from the local to the national. Thus, in the quote above from the publican about pub closures ‘destroying the whole fabric of British community’ the term community—which in this context might normally refer to the local neighbourhood that a particular pub serves—is given a national complexion, through the prefix ‘British’. Similarly, in an article in *The Huddersfield Daily Examiner* from 2012, the chief executive of the Campaign for Real Ale (CAMRA) is quoted as saying that ‘Whether situated in a small village, city high street or on the edge of a housing estate, pubs are so central to our society that whole communities can grow around a particular pub’. Here the geographical locality of community—village, high street or housing estate—is wedded to the larger, national scale of ‘our society’. References to pubs as part of the ‘social fabric’, ‘social and cultural fabric of the nation’ or ‘fabric of the British way of life’, were a common way in which this linking is achieved, with the term ‘fabric’ used in this manner 18 times in the 598 article sample. An article in *The Independent* from 2012 explicitly positions pubs as ‘part of the fabric that holds the country together’. Meanwhile an article in *The Daily Telegraph* from 2023 describes the loss of pubs as impacting the community‐spirit of the nation as a whole and as something of a national emergency, stating that:

…keeping Britain's once‐vibrant community spirit alive feels like an increasingly urgent task—and one we need to resolve by any means necessary.

This slippage between a local matter and a national issue appeared frequently in the sample. While it is not possible to deduce editorial motivation from content alone, we might infer that this was an important ‘hook’ used to engage an audience in a wider set of emotive concerns, with the imagined community of the British nation at its core.

Depictions of national community were more likely to be conjured through descriptions of some types of pubs over others. It is the ‘village pub’—and particularly the spectre of the pub‐less village—which is most often used to convey the damaging impact of pub closures on the ‘social fabric’ or the ‘fabric of the British countryside’. In the original 814 item sample, the word ‘village’ was the 13th most commonly used word, with 666 mentions. In contrast, ‘city’ was the 57th most common, with 316 mentions, and ‘town’ the 61st most common, with 312 mentions. Meanwhile in the 122‐article sample used for the qualitative analysis, the statistic that ‘less than half of British villages now have a local’ appeared in 3 articles in the 122‐article qualitative sample. In each case, the presentation of these statistics was accompanied by detailed reflections on the significance of pubs to British history. An article in *The Guardian* from 2006 headlined—‘*A carnage of local culture: The relentless closure of pubs by faceless property companies is an assault on our national heritage*’—describes the ‘uniquely pivotal role’ that pubs have played in British history and argues that they should be ‘preserved’ in the pursuit of ‘national identity and cultural integrity’. Meanwhile an article from 2008 in the *Huddersfield Daily Examiner* lauds the pub as ‘an institution which has lasted for more than 1000 years’, noting that the ‘pint’ measure in which beer is served was ‘ratified in the Magna Carta’ and that at the time of the Norman Conquest of 1066 ‘every hamlet had an alehouse’. Here, cultural scripts used to describe pubs (Markham 2024) support myths of national culture and identity rooted in a vision of a premodern, preurban English landscape, with the unchanging village pub or ‘hamlet alehouse’ providing a vital link to this primordial past.

Recent writing has focused on how contemporary pub closure, in a context of rising nationalism, has been represented as particularly affecting an English, working‐class constituency (Singh et al. 2024). As stated above, our content analysis of print media across a longer (23‐year) period did not find a focus on particular social groups as especially impacted by closure or being the cause of them. Rather, we find the inverse in the repeated efforts to present the pub in more universalising terms, as a cross‐class space of national community. The language of ‘social cohesion’ and, to a lesser extent, ‘cultural integration’ is important within these narratives. Often these terms are used without much explanation, but we get a sense of what might be meant in a longer, narrative piece in *The Guardian* by journalist John Harris. Writing during the Covid‐19 pandemic about what he calls the ‘everyday fellowship’ to be found in pubs, Harris describes observing:

…the mixing of generations gently enforced a certain set of social conventions. There was a sense that some people’s loneliness could at least be temporarily lifted by the crossword puzzles and small talk that defined the ‘early doors’ shift

Here, the social relations of the pub are presented as ameliorative of social problems and discontents. This goes beyond the potential benefits of easing feelings of loneliness and social isolation for vulnerable individuals (Thurnell‐Read 2021a) to extend pub culture as something central to the reproduction of the wider national community. Harris goes onto suggest that even moments of ‘excess and occasional violence’ characteristic of some pubs can be moderated by a ‘controlled environment’, one in which ‘these things were usually prevented from boiling over, and their deeper causes were sometimes even sorted out’. In Harris' narrative ‘Britain's pubs’, far from a space of exclusion and the preserve of one particular social group, operate as something like a stage, where social conflicts might somehow be reconciled.^2^

## 6 Discussion: The Banal Nationalism of Pub Closure Media Narratives

In the media texts analysed, the ubiquitous use of the definitive articles as *The* Local, *The* Pub or, indeed, *The* Great British Pub, serves to reify pubs as something homogeneous and unchanging. Presenting the nation ‘in homogeneous and unified terms’ is central to nearly all expressions of nationalism (May et al. 2020, 1056). Here this is achieved through repeated invocations of the pub as a site of community. Scaling from the local to the national, media narratives of pub closure present the pub as a kind of social stabiliser—without which the fabric of the nation might fall apart. While the sociological literature on pubs has focused mainly on the forms of face‐to‐face, in‐person community which pubs support (Dunbar 2016; Thurnell‐Read Forthcoming)—and the barriers to loneliness and forms of social capital and cohesion that this generates—we have drawn attention to the forms of ‘imagined community’ that media narratives of pub closure construct and the ways this operates as a form of banal nationalism. The ‘imagined community’ of the pub stands in for the ‘imagined community’ of the nation, one which ‘regardless of the actual inequality and exploitation that may prevail in each… is always conceived as a deep, horizontal comradeship’ (Anderson 1991, 7). Important here is how the pub is used to articulate a form of nationalism which, on the surface might transcend social divides through an emphasis on community and cohesion, in fact reproduces them by erasing the historical and contemporary exclusions associated with pubs in Britain and also by occluding an appreciation the diversity of pubs in Britain today. We therefore draw attention to the subtle ways in which the forms of normative community, belonging and non‐belonging, inherent to all forms of nationalism (Valluvan 2019), play out in this case.

In this final section, we critically reflect on these media narratives of pub closure, arguing that they work to occlude the ‘heterogeneity’ of pubs in Britain (Maye et al. 2005), and the varied ways pub closure is experienced. Specifically, they elevate the nation in a way that diminishes the possibility of a more nuanced, and certainly more sociologically interesting, treatment of the issue of pub closures. Thus, the nuances of the issue of pub closures, both in terms of the specific operational challenges facing the sector and the diversity of voices and experience that might otherwise be accounted for, are flattened out to heighten the appeal of a central and emotive framing of national loss.

While media narratives of pub closure tended to present the pub as inherently British, through the repetition of phrases such as ‘the great British pub’, there are a significant number of pubs in Britain where this assumption is, at the least, highly contestable. Thus, we might consider Irish pubs as sitting in ambivalent relation to the normative Britishness sustained across the narratives analysed. Part of the urban landscape in Britain since the end of the 19^th^ century, these offered Irish migrants to Britain a place to find work, hospitality and friendship in an exploitative and sometimes hostile host country (Tilki 2006). Likewise, the reified image of the British pub fails to acknowledge the Indian run ‘Desi pubs’ emerging as a significant yet until recently under‐appreciated space of cultural fusion to spread across Britain since the 1960s (Jesudason 2023). Often hosting ethnically mixed crowds of customers, Singh et al. (2024, 3) argue that Desi pubs are important spaces of ‘convivial multiculture’ in which ‘enduringly defensive taxonomies of race and nation’ give way to other, everyday forms of ‘cultural and social entanglement’.

The media texts tended to present the impacts of pub closure in terms of loss of ‘community’, with pubs portrayed as inclusive spaces in which people might form bonds of friendship with their neighbours, alleviate loneliness and create forms of social cohesion (See Thurnell‐Read 2021a). But, as discussed earlier, pubs have often been highly segregated spaces along lines of race, class, gender and sexuality—both internally with the provision of different rooms for men and women, and externally with non‐white people excluded from certain pubs in the post‐war period, up to as recently as the mid‐1990s (Jesudason 2023). Indeed, the presentation of ‘the pub’ as a place where class divisions either do not apply or are smoothed over overlooks the long‐established and ongoing distinction between ‘rough’ and ‘posh’ venues on the basis of social class (see M. A. Smith 1985; Nicholls 2019). For example, only one article in the sample highlights examples of closed estate pubs (pubs on social housing estates), despite these making up a sizeable proportion of British pubs.

Forgoing a nuanced examination of both the connections *and* divisions long present and still persistent in British pub culture, the concept of national community rendered in the media texts analysed tended to present an idealised, and simplified, image of pub communities and their associated ‘community spirit’. Instead, the tendency was to focus on ‘traditional’ pubs in idealised village communities or, to a lesser extent, examples of urban Victorian pub architecture—both of which can easily be made to represent ‘British’ pubs as a whole. They do not, however, account for the nuances of identity work and recursive boundary creation and reinforcement that take place on the ground within communities of pub goers (Goode and Anderson 2015; Watson and Watson 2012) and, in so doing, lean into and perpetuate the mythologies associated with the idealised British pub (Markham 2024).

It is worth noting that almost all the articles in the sample presented pub closure in negative terms. Poor quality pubs or lack of investment in pubs was posited as a cause of closure in only 4 articles (less than 1% of the sample), meaning historical concerns about the quality and character of pubs (Kneale 2021) received scant attention. The narratives identified offered little space for examining either the ‘changing priorities of many nightlife operators’ (Chatterton and Hollands 2002, 99) or the shift in consumer preferences from traditional pubs to other hospitality formats such as bars, restaurants and ‘hybrid’ café‐bars (Thompson et al. 2018). Neither did any articles address the potential positive health benefits of reduced rates of alcohol consumption that might be inferred from pub closures or the more general shift to more health conscious and risk adverse lifestyles, amongst young adults in particular (Yeomans et al. 2022).

That the great majority of print media coverage of pub closures over recent decades has done little to highlight and foster understanding of the complexities of what is a heterogeneous and highly diversified sector is, perhaps, the central function of these narrative framings. We suggest that the strong sympathy towards the issue of pub closure in the British print media is partly due to the status of pubs as symbols of the nation and ‘historically… masculine territory’ (Billig 1995, 119). A more nuanced picture may not be desired by either writer or reader of these texts.

Representations of the pub as site of idealised community have much in common with Orvell's (2012) analysis of the mythology of ‘Main Street’ in the United States. The central commercial street within a stereotypical American small town, Orvell argues that an idealised Main Street is central to dominant national imaginations of ordinary civic life in the United States. A ‘place of public spectacle’, Main Street is where people living in a neighbourhood come together and is ‘deeply tied up the American's desire for a perfect community’ which is insulated from contemporary dislocations and insecurities (Orvell 2012, 10). Like Main Street, the pub is both a banal feature of neighbourhoods across the country and a potent symbol of national culture in which notions of community are central. Media narratives of pub closure work across these different scales, connecting a crisis of community at the level of the neighbourhood to a story of national community in crisis. As Orvell states in the case of Main Street:

…in a society that is otherwise placeless and suffering from atopia and anomie…. Main Street stands for the rootedness of place and tradition.

(Orvell 2012, 11)

Similarly, we suggest that with British pubs the banal nationalism of pub closure media narratives project an idealised representation of community under threat. This gives shape to more generalised feelings of insecurity in the face of multiple crisis and dislocations faced by people in Britain today—some immediate and acute such as the pandemic^3^ and the ‘cost of living crisis’, others longer running, tied up with a long‐term stagnating economy, forms of deindustrialisation, changing gender relations and the gradual shift away from pubs as a primary site of leisure. Major changes in the landscape of consumer and retail space are also relevant here. As Yuill (2009, 2.2) argues regarding the collapse of the once ubiquitous high street chain Woolworths:

Abandoned space creates not just a spatial absence, but also an emotional absence. Much of the media and popular discourse surrounding Woolworths concerned not just the loss of a shop but also of supposed symbolic and emotional attachments.

Closed pubs can be read as part of a landscape of change and decline (Mah 2012; Thurnell‐Read 2021b). While narratives of entrepreneurial failure are sometimes lionised via heroic narratives (Ucbasaran et al. 2013) pub closure narratives in the British press almost exclusively invoke feelings of loss, sadness and regret. There is scope to suggest that narratives of loss, and the often‐evocative expressions of nostalgia bound up with them, are an increasingly prominent cultural trope. With this in mind, the ongoing media interest in pub closures and the zest with which the issue is bound up with a wider ‘threat to nation’ narrative might be read as a significant space in which the emotions, if not the practicalities, of this landscape of decline become animated and worked upon. As May et al. (2020), 1061 suggest, there is a need for sociology to continue to engage with the project of understanding how nationalism is ‘lived on the ground’ and, in this vein, we see here how the assumed everydayness of pubs and the social relations they support might be understood as allowing one particular avenue for navigating contemporary uncertainty and insecurity. Indeed, as Skey (2013) argues, the nation offers multiple forms of security—psychological and material—to those in the ethnic majority. Perhaps therefore the banal nationalism of pub closure media narratives provides its assumed audiences with something approximating the sense of community and belonging which is lamented as being under threat.

## 7 Conclusion

In this article, we argue that the narratives used to account for and communicate about the decades long decline of pub numbers invokes important cultural messages about nation and national culture. The history of the English pub is complex and has ebbed and flowed (Haydon 1994), yet framings of ‘loss’ often flatten this complex history and fail to account for the multiple and contested manifestations of pubs and pub culture. Rather, print media coverage of pub closures is shown to frequently invoke these closures as a loss of community and a threat to national identity.

Beyond the focus on media reporting of pub closures, the topic resonates with numerous contemporary sociological debates relating to the lived experiences of social and cultural change. While British pubs are culturally recognised nationally and internationally, we should be wary of slipping into a form of cultural exceptionalism. It is highly likely that similarly emotive debates about the actual or perceived loss or changes to valued social and cultural spaces in other countries, as Wang (2018) does in the case of teahouses in China. As such, the analysis presented here might prompt debates about closure, loss and nation beyond the admittedly specific case of British pubs. Indeed, while it is opportune that pubs, being an undervalued sociological topic (See Thurnell‐Read 2021b), provide a particularly evocative example of how sentiments of loss and threat relating to national identity become animated, the significance of these insights may resonate with a wider set of sociological concerns.

It is important to acknowledge several limitations of the research. Notably, while we infer a preference for a simplified telling of the story of pubs closures as something unified and bridging of the great many complexities and disconnections ‘on the ground’, media content analysis alone does not allow us to access to journalists and editors to confirm this as intentional and desired. Audience response is also inferred, meaning that while we identify recurring emotive narratives linking the loss of pubs to a threat to national identity, we are left again inferring the emotional response of readers of such texts. Likewise, the media content analysed represents only a small and admittedly particular fragment of the many locations in which the practicalities of pub closures and the sentiments associated with their loss are animate. Indeed, the ways pub closure is experienced and narrated in particular communities is central to our ongoing research in this area.

## Conflicts of Interest

The authors declare no conflicts of interest.

## Data Availability

Raw data were generated through the Nexis database. Derived data supporting the findings of this study are available from the corresponding author RD on request.

## Endnotes

^1^
The pub in Coronation Street –‘The Rovers Return’—temporarily closed in 2023 amid a ‘cost of living crisis’.

^2^
This recalls Kate Fox's (2004) pop‐anthropological analysis of the minutia of pub etiquette as one, if not *the*, shibboleths of English culture.

^3^
See O'Brien (2021) for an analysis of the use of public houses as a collective representation of Irish society during the covid‐19 pandemic.
